# 超声气管镜针吸活检在肺癌诊断及基因检测中的作用

**DOI:** 10.3779/j.issn.1009-3419.2018.09.04

**Published:** 2018-09-20

**Authors:** 闽江 陈, 池 邵, 燕 徐, 雪峰 孙, 静 赵, 勇 陈, 媛媛 赵, 巍 钟, 孟昭 王

**Affiliations:** 100730 北京，中国医学科学院，北京协和医学院，北京协和医院呼吸内科 Department of Respiratory Medicine, Peking Union Medical College Hospital, Chinese Academy of Medical Sciences and Peking Union Medical College, Beijing 100730, China

**Keywords:** 超声气管镜针吸活检, 肺肿瘤, 基因检测, Endobronchial ultrasound guided transbronchial needle aspiration, Lung neoplasms, Gene analysis

## Abstract

**背景与目的:**

超声气管镜针吸活检（endobronchial ultrasound guided tranbronchial needle aspiration, EBUS-TBNA）是临床怀疑肺癌患者的常用活检方式，在肺癌的诊断和分期中有着举足轻重的作用。然而该活检方式在诊断之余是否亦能提供充分的组织完成基因检测尚待研究。本文评价EBUS-TBNA所取得标本进行肺癌诊断及相关基因检测的可行性。

**方法:**

对纵隔淋巴结肿大且临床怀疑肺癌诊断的患者进行EBUS-TBNA活检，所取得的标本进行病理诊断并对其中的非鳞非小细胞肺癌标本进行驱动基因检测。分析其诊断阳性率以及完成基因检测的可行性。

**结果:**

入选377例患者平均单个淋巴结穿刺2.07针，确诊肺癌213例，经EBUS-TBNA诊断率92%。其中表皮生长因子受体（epidermal growth factor receptor, *EGFR*）基因、间变淋巴瘤激酶（anaplasticlymphoma kinase, *ALK*）融合基因、以及同时完成两个基因检测的患者分别为84例（90%）、105例（96%）及79例（90%）。单因素分析显示组织基因检测成功率与穿刺淋巴结针数、淋巴结大小及淋巴结部位无关，但与肿瘤病理类型相关。腺癌病理类型的*EGFR*基因突变及*ALK*融合基因检测的成功率均高于未分类非小细胞肺癌。

**结论:**

EBUS-TBNA可提供充足的组织对肺癌进行诊断和基因分型。肿瘤病理类型可能是影响基因检测阳性率的因素。

## Abstract (en)

**Background and objective:**

Endobronchial ultrasound guided transbronchial needle aspiration (EBUS-TBNA) has emerged as an innovative technique for diagnosis and staging of lung cancer. But whether the procedure can provide enough tissue for the detection of gene mutations is still to be defined. Here we evaluated the efficacy of lung cancer diagnosis and gene analysis using samples obtain via EBUS-TBNA.

**Methods:**

Patients with suspected lung cancer and mediastinal lesions were referred for EBUS-TBNA. Diagnosis and sub-classifications were made by pathologists. Samples with non-squamous non small cell lung cancer sub type were tested for the *EGFR* and/or *ALK* mutations.

**Results:**

A total of 377 patients were included in this study. The median needle passes were 2.07. Lung cancer was diagnosed in 213 patients. The diagnosis accuracy for malignancy was 92%. Epidermal growth factor receptor (*EGFR*) mutations, anaplasticlymphoma kinase (*ALK*) fusion genes and double genes analysis were successfully preformed in 84 (90%), 105 (95%) and 79 (90%) patients. The number of needle passes and the diameters of lymph node were not associated with the efficacy of gene testing in univariate analysis. However, samples of adenocarcinoma sub type showed a tendency associated with higher genotyping efficacy.

**Conclusion:**

Tissue samples obtained through EBUS-TBNA are sufficient for pathological diagnosis and genetic analysis of lung cancer. The pathology type of sample affected genotyping efficacy.

肺癌是最常见导致死亡的恶性肿瘤^[1]^，发病时大多数患者即存在转移。纵隔淋巴结活检对明确诊断和分期至关重要，在部分患者中可能是唯一的病理诊断和分期依据。既往纵隔病变主要依靠纵隔镜或胸腔镜进行活检，但创伤大，成本高。超声气管镜引导下针吸活检（endobronchial ultrasound guided tranbronchial needle aspiration, EBUS-TBNA）可在超声实时引导下对大气道外的纵隔病灶和淋巴结进行活检，是一项高效微创的检查手段，且诊断准确性高和并发症发生率低，在肺癌诊断和分期中得到了广泛应用^[2, 3]^。

近年来，肺癌特别是非小细胞肺癌的个体化治疗飞速发展，这对病理诊断和活检组织的充分性提出了更高的要求。对于晚期非小细胞肺癌患者，驱动基因突变检测是指导治疗的一个重要条件。对于存在基因突变的患者，应用靶向治疗相对于化疗疗效更佳且毒性作用更低，因此，已经在各项国际和国内指南中被列为一线治疗的首选方案。EBUS-TBNA所取得的活检组织作为一种小标本，可否满足临床对于基因检测的需求，是临床医生关注的重要问题。

本研究对北京协和医院呼吸科单中心接受EBUS-TBNA检查的肺癌患者进行分析，探讨EBUS-TBNA标本进行肺癌诊断和驱动基因检测的可行性。

## 1 资料与方法

### 1.1 入选患者

收集北京协和医院呼吸科自2013年12月-2016年12月临床考虑为肺癌并进行了EBUS-TBNA检查的患者。入选条件为包括：年龄 > 18岁；电子计算机断层扫描（computed tomography, CT）或正电子发射计算机断层显像（positron emission tomography, PET）/CT提示纵隔占位性病变或纵隔淋巴结肿大；临床怀疑为胸部恶性肿瘤；接受EBUS-TBNA检查。收集患者的年龄、性别、纵隔淋巴结分组和大小、EBUS-TBNA穿刺情况（包括穿刺部位、穿刺针数、不良反应）、最终病理诊断以及分子检测结果。

### 1.2 操作方法

检查在局麻或静脉中等程度静麻醉下进行。首先进行常规电子气管镜（Olympus BF-260）检查观察管腔，如可发现明确新生物，则进行活检；如无明确新生物，或预计活检标本不理想，则换用EBUS（超声气管镜：OlympusUC-260-FW；超声主机：Olympus EU-ME1）对影像提示的纵隔病灶依次进行超声检查，选择拟穿刺的淋巴结，并在超声引导下应用22 G穿刺针（一次性细胞学穿刺针：22 G-NA-201SX-4022）对目标淋巴结进行穿刺活检。穿刺以获得明确组织条或4针后停止。对于同时需要穿刺不同站淋巴的患者，依据淋巴结转移可能性由对侧至同侧纵隔肺门淋巴结依次穿刺并分别送病理。经穿刺取得的组织标本经福尔马林固定24 h后石蜡包埋切片，穿刺细胞学标本涂片后应用95%乙醇固定，支气管肺泡灌洗标本由病理科经离心后涂片固定。对临床诊断不除外感染的患者在淋巴结穿刺后进行穿刺针冲洗涂片及培养。病理诊断均由病理专科医生出具正式报告。

### 1.3 诊断标准

患者最终的诊断确定包括：①恶性肿瘤：必须为病理学诊断，标本来源包括EBUS-TBNA，也包括其他方法，如CT引导下穿刺、胸水细胞学、淋巴结活检或手术等；②结节病：诊断参照中华结核和呼吸杂志报道的结节病诊断标准；③肺结核：有明确的抗酸染色阳性或上皮样肉芽肿伴干酪样坏死；④非特异性炎性淋巴结：抗生素治疗后明显缩小或1年随访无增大。

### 1.4 免疫组化及基因检测

对于常规染色不能确定病理类型的患者进行TTF-1、CK、CEA、P40、P63、Syn等组合进一步确定病理分型。对于最终病理为非鳞非小细胞肺癌的患者加送表皮生长因子受体（epidermal growth factor receptor, *EGFR*）基因突变及间变淋巴瘤激酶（anaplasticlymphoma kinase, *ALK*）融合基因检测。*EGFR*基因突变采用突变扩增阻滞系统聚合酶链式反应（amplification refractory mutation system-polymerase chain reaction, ARMs-PCR，试剂盒theascreen EGFR RGQ PCR Kit），对于肿瘤标本要求组织肿瘤细胞数大于100个，经提取后肿瘤组织核酸浓度满足PCR上机要求者再进行检测。*ALK*基因应用原位免疫荧光杂交（FISH）方法或D5F3抗体免疫组化方法进行检测，对于每个病例均设立阳性和阴性对照。FISH方法（试剂盒Vysis LSI ALK Dual Color, Break Apart Rearrangement Probe; Abbott Molecular, Rungis, France）需计数至少50个肿瘤细胞中大于15%细胞呈现分离的红色或绿色信号或单纯出现红色信号提示为阳性结果。

### 1.5 统计方法

应用SPSS 17.0软件对数据进行处理。计算诊断的有效率、敏感性、特异性、阳性预测值、阴性预测值和组间差异。

## 2 结果

### 2.1 患者基本情况

本组共377例患者进行了EBUS-TBNA检查，其中位年龄58岁，男性213例，女性164例。377例患者共穿刺淋巴结420组，共计868针，平均单个淋巴结穿刺2.07针。经EBUS-TBNA取得病理诊断的肺癌213例，其中腺癌90例（42%），未分类的非小细胞肺癌39例（18%），鳞癌24例（11%），小细胞肺癌60例（28%）。经随访及其他活检方式诊断假阴性淋巴结30组，假阴性患者26例。不同区域淋巴结和不同疾病的EBUS-TBNA诊断准确性见表 1和表 2。

**1:** 穿刺部位及诊断率 Mediastinal lymph node stations and diagnosis accuracy by stations

Lymph node stations	Lymph node number	Needle passes/Median	Lung cancer	Fasle negative	Accuracy (%)
N2	7/2%	14/2.00	5	1	86
N4	124/30%	243/1.96	73	4	97
N5	5/1%	10/2.00	4	1	80
N7	184/44%	390/2.12	82	15	92
N10	42/10%	91/2.17	21	1	98
N11	31/7%	58/1.87	18	2	94
Mediastinal mass	27/6%	62/2.30	15	5	81
Total	420	868/2.07	218	30	93

**2:** 最终病理诊断 Pathology diagnosis

Pathology diagnosis	Number	Diagnosed by EBUS	False diagnosis by EBUS	Accuracy (%)
Non-small cell lung cancer	170	153	17	90
Adenocarcinoma	98	90	8	92
Squamous carcinoma	25	24	1	96
Not otherwise specified	47	39	8	83
Small cell lung cancer	62	60	2	97
Sarcoidosis	39	35	4	90
Tuberculosis	17	16	1	94
Metastasis tumor	3	3	0	100
Benign/reactive nodes	86	110	2	80
EBUS: endobronchial ultrasound.				

EBUS-TBNA诊断的216例肿瘤患者中，共有203（94%）例患者的标本进行了必要的免疫组化染色，其中199例经免疫组化染色后有明确分型诊断。

### 2.2 非鳞非小细胞肺癌患者基因检测情况

经EBUS-TBNA诊断的非鳞非小细胞肺癌共129例，包括腺癌90例和未分类的非小细胞肺癌39例。其中93例申请了ARMs法*EGFR*基因突变检测，其组织足以进行检测的患者为84例（90%），组织标本不足以进行检测的9例（10%）。最终检测结果显示*EGFR*突变阳性的患者为44例（52%），其中19外显子缺失突变25例（57%），21外显子21L858R点突变17例（41%），另外检测到21L861Q突变1例和18 G719X突变1例。

其中111例患者进行了*ALK*融合基因检测，其中19例应用FISH方法进行检测，92例应用免疫组化方法进行检测。应用FISH方法的检测成功率为89%，应用免疫组化方法检测的成功率为96%，两种方法检测阳性患者分别为1例（6%）和5例（6%）。

部分患者还接受了其他的基因检测，包括二代测序3例、c-met免疫组化检测2例、*ROS*-*1*融合基因3例及*KRAS*突变检测7例。上述患者中，同时联合*EGFR*和*ALK*融合基因检测患者共87例，其中检测均成功的为79例（90%）。

### 2.3 EBUS-TBNA组织基因检测成功的影响因素

组织是否充足是基因检测能否成功进行的重要因素。本研究对可能影响组织充分性，导致检测不成功的因素进行了分析，结果表明，穿刺针数、淋巴结直径和淋巴结部位和与检测成功率无明显相关。腺癌病理类型较非腺癌类型的患者基因检测成功率高，差异有统计学意义（*P* < 0.05）（表 3-表 5）。

**3:** 影响*EGFR*基因检测成功率的因素 Factors affect EGFR genotyping efficacy

	Successful	Unsuccessful	*P*
Needle passes (Mean±SD, *n*)	2.17±0.60	2.44±0.88	0.405
Lymph node diameter (Mean±SD, cm)	2.39±1.10	2.27±1.00	0.932
Lymph node stations			
N4	23	5	0.122
N7	42	2	0.164
Others	19	2	1.000
Pathology			
Adenocarcinoma	71	3	0.000
Others	13	6	
EGFR: epidermal growth factor receptor			

**4:** 影响*ALK*基因检测成功率的因素 Factors affect ALK genotyping efficacy

	Successful	Unsuccessful	*P*
Needle passes (Mean±SD, *n*)	2.16±0.61	2.00±0.50	0.442
Lymph node diameter (Mean±SD, cm)	2.37±0.83	2.90±0.87	0.979
Lymph node stations			
N4	32	1	1.000
N7	36	1	0.646
Others	20	2	0.241
Pathology			
Adenocarcinoma	72	1	0.006
Others	16	3	

**5:** 影响*EGFR*+*ALK*基因检测成功率的因素 Factors affect double genotyping efficacy

	Successful	Unsuccessful	*P*
Needle passes (Mean±SD, *n*)	2.16±0.64	2.00±0.50	0.492
Lymph node diameter (Mean±SD, cm)	2.79±1.15	2.27±0.94	0.192
Lymph				
N4	26	3	1.000
N7	35	1	0.133
Others	18	4	0.107
Pathology				
Adenocarcinoma	68	3	0.001
Others	11	5	

### 2.4 术前麻醉方式、操作不良反应及并发症

本研究中患者主要接受的麻醉方式为局麻，其中325例（86%）为局麻下操作。所有接受EBUS-TBNA的患者均未出现死亡、大咯血、支气管瘘等严重并发症。常见的并发症状包括操作后咳嗽、咳痰、少量咯血及低热，均无需住院治疗。

## 3 讨论

对于临床怀疑胸部恶性肿瘤且合并纵隔淋巴结肿大的患者来说，取得足够的病理进行诊断尤其重要。微创的活检方式包括气管镜、CT引导下经皮肺穿刺活检以及近年来逐渐进入使用的EBUS-TBNA。其中后者对于气管腔外的病灶能在超声引导下充分取材，与同为微创取材方式的CT引导下经皮肺穿刺活检相比，更加有利于肿瘤分期以及反复操作取得的标本，且并发症更少^[4]^。因此，EBUS-TBNA广泛用于肺癌的分期及纵隔疾病的诊断^[5, 6]^。准确性方面根据之前报道的结果，与纵隔疾病诊断的金标准纵隔镜相比，EBUS-TBNA可达到92%的诊断准确率，其中对于肺癌的诊断准确率可高达97%^[7]^。因此EBUS-TBNA在美国国立综合癌症网络（National Comprehensive Cancer Network, NCCN）非小细胞肺癌指南^[8]^中被推荐作为肺癌术前分期的重要手段。

本研究对本中心377例临床怀疑肿瘤的患者进行了分析和随访，最终经EBUS-TBNA的诊断率与之前报道相仿，提示EBUS-TBNA对于怀疑胸部肿瘤的患者的明确诊断是一种良好的检查手段。此外，本研究结果提示不同穿刺部位的阳性率不同，其中上气管旁淋巴结（N2组）、主肺动脉窗淋巴结（N5组）以及纵隔肿物穿刺阳性率相对较低，考虑可能与上述部位的淋巴结穿刺相对困难相关、非常规穿刺取材部位（N5组淋巴结）^[9]^、以及穿刺定位标志不明确相关。不同组织类型的肺癌中，小细胞肺癌的诊断率最高，而非小细胞未分化癌的诊断率相对较低。考虑与小细胞肺癌容易表现为纵隔淋巴结转移的生物学行为相关。同时，非小细胞未分化癌往往由于分化程度更低，免疫组化无典型表现增加了诊断的难度，故诊断率相对较低。

近年来，国际肿瘤协会对于肺癌的分型分类也进行了更新。在小标本诊断中，更需要应用免疫组化区分具体病理类型以指导治疗。因此，对于肺癌患者，取得活检组织不仅需要能够提供肿瘤的病理诊断，还需要提供具体分型的信息。Navavi等^[10]^报道了774例小标本患者进行免疫组化染色后进行分型，减少了非小细胞肺癌组织不明确型的诊断率，也提示了免疫组化对于肺癌分型的重要性。在本研究中经EBUS-TBNA诊断的216例肿瘤患者中，94%标本进行了免疫组化染色，并明确分型诊断。提示EBUS-TBNA取得的标本对于分型诊断的是充足的。

肺癌个体化治疗的进展还体现在靶向药物的广泛应用。对于具有基因突变的晚期非小细胞患者，应用靶向治疗可延长患者的无进展生存时间且副作用更小^[11, 12]^。因此，*EGFR*基因突变以及*ALK*融合基因检测目前已被NCCN指南推荐为晚期非小细胞肺癌的常规检查项目。因此，应用EBUS-TBNA取得的组织是否能足以进行基因检测也引了越来越多的关注。之前报道应用EBUS-TBNA组织进行*EGFR*基因检测的成功率为72%-97%不等^[13-15]^，*ALK*融合基因的检测成功率为71%-95%（FISH方法）及近100%（免疫组化方法）^[16, 17]^。但这些研究的病例数均较少，且大多来自非亚裔人群，本身突变比例较低，不能很好地反映中国人群进行EBUS-TBNA取材并进行检测的状况。

目前，*EGFR*基因突变的检测方法包括DNA测序法、PCR法以及二代测序等^[18, 19]^。本研究中应用的检测方法为ARMS法，是一种敏感性和特异性均高的检测手段^[20]^。本组患者中共有93例进行了*EGFR*基因突变检测，检测成功率为90%，与之前报道相似。与我院近年来经气管镜粘膜活检标本进行基因检测的成功率相比，EBUS-TBNA所取得标本的检测成功率更高（分别为90%和85%）^[21]^。提示EBUS-TBNA与粘膜活检相比可提供更充足的组织进行基因检测。

针对*ALK*融合基因检测，目前NCCN指南^[8]^推荐的标准检测方法为FISH方法，但也同时提出应用应用免疫组化方法可以作为*ALK*融合基因的筛查手段。近年来，多项研究也表明应用免疫组化方法的敏感性和特异性可与FISH方法相当^[22-25]^。在Rogers等^[24]^的研究中提出，应用D5F3抗体进行免疫组化，检测*ALK*融合基因的敏感性可达100%。由于操作更加简单，对标本量的要求更低，我院目前进行的*ALK*融合基因检测主要采用D5F3抗体的免疫组化方式。在本研究中进行ALK检测的患者，其中19例进行了FISH检测，其他应用免疫组化的检查方式，应用FISH方法和免疫组化方法检测的成功率分别为89%和98%，最终检测的阳性率为6%，与之前报道相当。提示应用EBUS-TBNA对于*ALK*融合基因的检测也能提供充足的组织标本。

此外，患者中进行联合两项基因检测组织充足可以完成检测的比例为92%，进行三项检测检且组织充足的为58%，提示EBUS-TBNA对肺癌患者的基因分型可提供充足的组织标本。

影响基因检测成功率的因素众多，包括组织充分性因素以及检测相关的因素。本研究对EBUS操作因素和进行基因检测的组织充分性关系进行了分析，结果提示穿刺针数、进行穿刺操作的淋巴结部位和大小不影响进行基因检测的组织充分性。考虑可能与所有患者均接受较多穿刺数（平均2.07针）相关，同时也提示或许不需要为行基因检测而对肺癌患者进行更多次的穿刺操作且对于CT发现的纵隔小淋巴结只要是EBUS-TBNA可穿刺部位，均有可能经EBUS-TBNA穿刺取得足够进行病理检测的组织。此外，不能分类非小细胞因组织不足导致的基因检测失败率更高，可能与不能分类的非小细胞癌分化更差，需要更多组织切片进行多项免疫组化染色相关。

可行性及安全性方面，本研究中的大多数患者为局麻下操作，且无人出现严重并发症。从另一方面说明了EBUS-TBNA是一项便于操作且安全有效的检查手段。

本研究的不足之处在于因经济及变更诊治地点等因素，部分患者未能进行基因检测。此外，进行3项以上基因检测，以及进行*KRAS*、*CMET*以及*ROS*-*1*等基因检测的患者例数较少。今后研究中可前瞻性对所有符合要求的标本进行多个基因检测以充分评估EBUS-TBNA组织用于多基因检测的充分性。

综上所述，EBUS-TBNA是一种安全有效的检查手段。其取得标本可满足非小细胞肺癌患者进行包括*EGFR*以及*ALK*融合基因在内的多项不同类型的基因检测需要，可作为伴有纵隔淋巴结转移的晚期非小细胞肺癌患者的活检手段之一。肿瘤的病理类型是影响检测成功率的可能因素。
